# Artificial Thermostable D-Amino Acid Dehydrogenase: Creation and Application

**DOI:** 10.3389/fmicb.2018.01760

**Published:** 2018-08-03

**Authors:** Hironaga Akita, Junji Hayashi, Haruhiko Sakuraba, Toshihisa Ohshima

**Affiliations:** ^1^Research Institute for Sustainable Chemistry, National Institute of Advanced Industrial Science and Technology (AIST), Hiroshima, Japan; ^2^Department of Biotechnology, College of Life Sciences, Ritsumeikan University Biwako-Kusatsu Campus, Shiga, Japan; ^3^Department of Applied Biological Science, Faculty of Agriculture, Kagawa University, Kagawa, Japan; ^4^Department of Biomedical Engineering, Faculty of Engineering, Osaka Institute of Technology, Osaka, Japan

**Keywords:** D-amino acid, thermostable D-amino acid dehydrogenase, *meso*-diaminopimelate dehydrogenase, stable isotope-labeled D-amino acid, *Ureibacillus thermosphaericus*, protein engineering, crystal structure analysis

## Abstract

Many kinds of NAD(P)^+^-dependent L-amino acid dehydrogenases have been so far found and effectively used for synthesis of L-amino acids and their analogs, and for their sensing. By contrast, similar biotechnological use of D-amino acid dehydrogenase (D-AADH) has not been achieved because useful D-AADH has not been found from natural resources. Recently, using protein engineering methods, an NADP^+^-dependent D-AADH was created from *meso*-diaminopimelate dehydrogenase (*meso*-DAPDH). The artificially created D-AADH catalyzed the reversible NADP^+^-dependent oxidative deamination of D-amino acids to 2-oxo acids. The enzyme, especially thermostable one from thermophiles, was efficiently applicable to synthesis of D-branched-chain amino acids (D-BCAAs), with high yields and optical purity, and was useful for the practical synthesis of ^13^C- and/or ^15^N-labeled D-BCAAs. The enzyme also made it possible to assay D-isoleucine selectively in a mixture of isoleucine isomers. Analyses of the three-dimensional structures of *meso*-DAPDH and D-AADH, and designed mutations based on the information obtained made it possible to markedly enhance enzyme activity and to create D-AADH homologs with desired reactivity profiles. The methods described here may be an effective approach to artificial creation of biotechnologically useful enzymes.

## Introduction

With the exception of glycine, proteinogenic α-amino acids all contain an asymmetrical carbon, resulting in the occurrence of D- and L-enantiomers. However, high enantiomeric purity is necessary for a variety of industrial applications in biotechnological fields. The greatest demand is currently for L-amino acids. Indeed, several million tons of L-amino acids are utilized annually in animal feed (Becker and Wittmann, 2012), food supplements (Becker and Wittmann, 2012), pharmaceuticals (Ivanov et al., 2013), and other applications (Leuchtenberger et al., 2005). On the other hand, D-amino acids have been increasingly utilized in the recent years, principally for industrial and pharmaceutical applications. For example, the artificial sweeter alitame is composed of a dipeptide of D-alanine and L-aspartic acid (Chattopadhyay et al., 2014). In addition, D-phenylglycine and D-*p*-hydroxyphenylglycine are used as core building blocks of semisynthetic cephalosporins and penicillins (Martínez-Rodríguez et al., 2010), and annual production amount of their peptides has reached 2,500 and 60,000 tons, respectively (Silber et al., 2016).

D-Amino acids are produced mainly through chemical and enzymatic syntheses. Strecker synthesis is a convenient method of chemical synthesis. However, enantioselectivity is not high with this method, and subsequent purification steps are required to obtain pure D-amino acids. Enzymatic synthesis is more suitable for synthesis of D-amino acids with higher enantioselectivity. As a current industrial scale-production method, D-hydroxyphenylglycine production using two coupled reactions with D-hydantoinase and D-carbamoylase is the most popular. Using this system, several thousand tons of D-hydroxyphenylglycine is produced annually (Schmid et al., 2002). In addition, other methods using aminoacylase (Wakayama et al., 2003) and serine aldolase (Araki et al., 2011) have been developed for the industrial scale production of several D-amino acids and D-serine, respectively. As an alternative enzymatic method of D-amino acid production, the use of reductive amination of 2-oxo acids using NAD(P)^+^-dependent amino acid dehydrogenase is anticipated. About 20 types of amino acid dehydrogenase have been identified to date, but nearly all are L-amino acid-specific and are used for L-amino acid production from oxo acids and ammonia (Ohshima and Soda, 2000). One exception, *meso*-diaminopimelate dehydrogenase (EC1.4.1.16, *meso*-DAPDH), catalyzes the reversible deamination of a D-amino acid moiety in *meso*-2,6-diaminopimelate (*meso*-diaminopimelate) to L-2-amino-6-oxopimelate in the presence of NADP^+^ (Misono et al., 1979) This means that the enzyme is a kind of NADP^+^-dependent D-amino acid dehydrogenase (D-AADH), and it was expected to be applicable to synthesis of *meso*-diaminopimelate and analogs through amination of oxo acids. However, the enzyme exhibits extremely high specificity to *meso*-diaminopimelate (Misono and Soda, 1980) and will not catalyze the amination of 2-oxo acid to D-amino acid because the reaction product L-2-amino-6-oxopimelate is cyclized *in vitro*. Consequently, attempts to utilize this enzyme for D-amino acid synthesis were abandoned. In the meantime, Vedha-Peters et al. (2006) succeeded in creating a D-AADH from the starting enzyme *Corynebacterium glutamicum meso*-DAPDH through introduction of five point mutations using both rational and random mutagenesis and screening. They showed that the engineered enzyme was able to produce several D-amino acids through reductive amination of the corresponding 2-oxo acids. However, this D-AADH prepared from mesophilic *C. glutamicum meso*-DAPDH was not sufficiently stable for use as the catalyst in a bioreactor. A more stable D-AADH with high catalytic activity was needed for its practical uses.

To overcome the aforementioned drawbacks, we initially used genome information to look for gene homologs of *meso*-DAPDH in large number thermophilic microorganisms, but did not find it. We next tried screening a large series of thermophilic microorganisms for *meso*-DAPDH activity and found it in a thermophile isolated from Japanese compost (Akita et al., 2011). The thermophile was identified as *Ureibacillus thermosphaericus* strain A1, from which we were able to purify a thermostable *meso*-DAPDH. We then characterized the enzymatic property and determined its gene’s sequence. This in turn enabled us to create stable D-AADHs through designed mutations of *meso*-DAPDH and to develop a useful new bioreactor using D-AADH for the production of D-amino acids, including stable isotope-labeled D-amino acids (Akita et al., 2012). Based on structural analyses of *meso*-DAPDH and D-AADH (Akita et al., 2015), D-AADH function was also upgraded (Hayashi et al., 2017). A little later in our study, another thermostable *meso*-DAPDH from an uncultivable thermophile, *Symbiobacterium thermophilum* IAM14863 was cloned, produced in *Escherichia coli*, and characterized (Gao et al., 2012). This *S. thermophilum* thermostable *meso*-DAPDH has been reported to exhibit somewhat unusual substrate specificity compared with those of *C. glutamicum* and *U. thermosphaericus* counterparts. After that, structural and mutational studies on *S. thermophilum* and *Clostridium tetani meso*-DAPDHs followed to obtain new types of D-AADH (Liu et al., 2014, 2015). In this review, the creation of novel thermostable D-AADHs and their useful application are described focusing on our recent research results.

## Screening for a Thermostable *meso*-DAPDH

Bacterial biosynthetic pathways for L-lysine are classified into three slightly different pathways: acetylase, dehydrogenase, and succinylase pathways (Born and Blanchard, 1999). *meso*-DAPDH functions in the dehydrogenase pathway and catalyzes the reversible NADP^+^-dependent oxidative deamination of *meso*-diaminopimelate to produce L-2-amino-6-oxopimelate. A key feature of *meso*-DAPDH is its ability to recognize the difference between D- and L-configurations of *meso*-diaminopimelate (Scapin et al., 1998). The enzyme acts specifically on *meso*-diaminopimelate, though not the DD- or LL-forms. In addition, the enzyme acts on only the D-amino acid center of *meso*-diaminopimelate, not the L-amino acid center. *meso*-DAPDHs have been identified in mesophilic bacteria, including *Bacillus sphaericus* (Misono et al., 1979; Misono and Soda, 1980), *Brevibacterium* sp. (Misono et al., 1986b), and *C. glutamicum* (Misono et al., 1986a). Among them, *meso*-DAPDH from *C. glutamicum* has been characterized in detail and the three-dimensional structures of the enzyme/NADP^+^ complex (Scapin et al., 1996), the enzyme/substrate complex, and the enzyme/NADP^+^-inhibitor complex (Scapin et al., 1998) have been solved. Moreover, Vedha-Peters et al. (2006) prepared an NADP^+^-dependent D-AADH by introducing five substitutions at the active site of the *C. glutamicum meso*-DAPDH. This mutant enzyme is capable of one-step production of several L-amino acids via reductive amination of the corresponding 2-oxo acids with ammonia and NADPH. However, because this enzyme was prepared from a mesophilic bacterial *meso*-DAPDH, it is not sufficiently stable for use under the conditions necessary for industrial application. A more stable *meso*-DAPDH and mutant enzymes are required for biotechnological application.

To obtain those more stable enzymes, we first focused on detecting homologs of thermostable *meso*-DAPDH in a database, as we expected that thermostable enzymes would be much more stable than mesophilic ones. Unfortunately, we found no *meso*-DAPDHs from thermophiles or gene homologs from a starting point around 2010. We next screened for *meso*-DAPDH activity in more than 100 thermophilic strains isolated from various environmental samples. We eventually detected an enzyme produced in a thermophile isolated from compost collected in Munakata City, Fukuoka Prefecture, Japan. Based on 16S rRNA gene sequence similarity, the isolated strain was identified as *U. thermosphaeicus* strain A1 (Akita et al., 2011). After optimizing the culture conditions for growth of *U. thermosphaeicus, meso*-DAPDH was purified from the strain through five successive chromatography steps. As expected, the purified enzyme was much more thermostable than mesophilic ones, and showed almost no loss of activity after incubation for 30 min at 60°C. By contrast, nearly all the activity of the *C. glutamicum* enzyme was lost after incubation for 10 min at 48°C (Misono et al., 1986a). Moreover, the *U. thermosphaericus* enzyme was stable at pH 5.0 to 11.0, even after incubation for 30 min at 50°C (Akita et al., 2011), which suggests the *meso*-DAPDH from *U. thermosphaericus* could be a useful source for creation of sufficiently stable D-AADHs.

To determine the *U. thermosphaericus meso*-DAPDH gene sequence, the *N*-terminal amino acid sequence of the enzyme was determined. With that sequence information, degenerate primers were designed, and partial amplification of the *meso*-DAPDH gene was performed using the degenerate primers. Finally, the complete sequence (981 bp) of the *meso*-DAPDH gene was determined using an *in vitro* Cloning Kit (Takara Bio, Shiga, Japan), and the encoded *N*-terminal amino acid sequence was identical to that obtained through protein sequencing (Akita et al., 2011). From multiple sequence alignment of *meso*-DAPDH genes from *U. thermosphaericus* and several other bacterial strains, we found that the five amino acid residues (Gln150, Asp154, Thr169, Arg195, and His244) mutated in the *C. glutamicum* enzyme to create a D-AADH were completely conserved in the sequence of the *U. thermosphaericus* enzyme.

Based on the similar concept, Gao et al. (2012) found *meso*-DAPDH gene homolog in an uncultivable thermophile, *S. thermophilum*, and succeeded in its expression in *E. coli* and characterization of *meso*-DAPDH as the second thermostable enzyme. The substrate specificity of this *meso*-DAPDH is different from those of *meso*-DAPDHs from other sources including *C. glutamicum* and *U. thermosphaericus*; *S. thermophilum meso*-DAPDH catalyzes the reversible reductive amination of D-alanine, D-valine and D-lysine as well as *meso*-diaminopimelate. In addition, the hexameric structure of *S. thermophilum meso*-DAPDH is clearly different from the dimeric structure of *C. glutamicum* and *U. thermosphaericus* enzyme. Such relaxed substrate specificity may be effective for the creation of alternative D-AADH by protein engineering.

## Creation of Thermostable D-AADH

**FIGURE 1:**
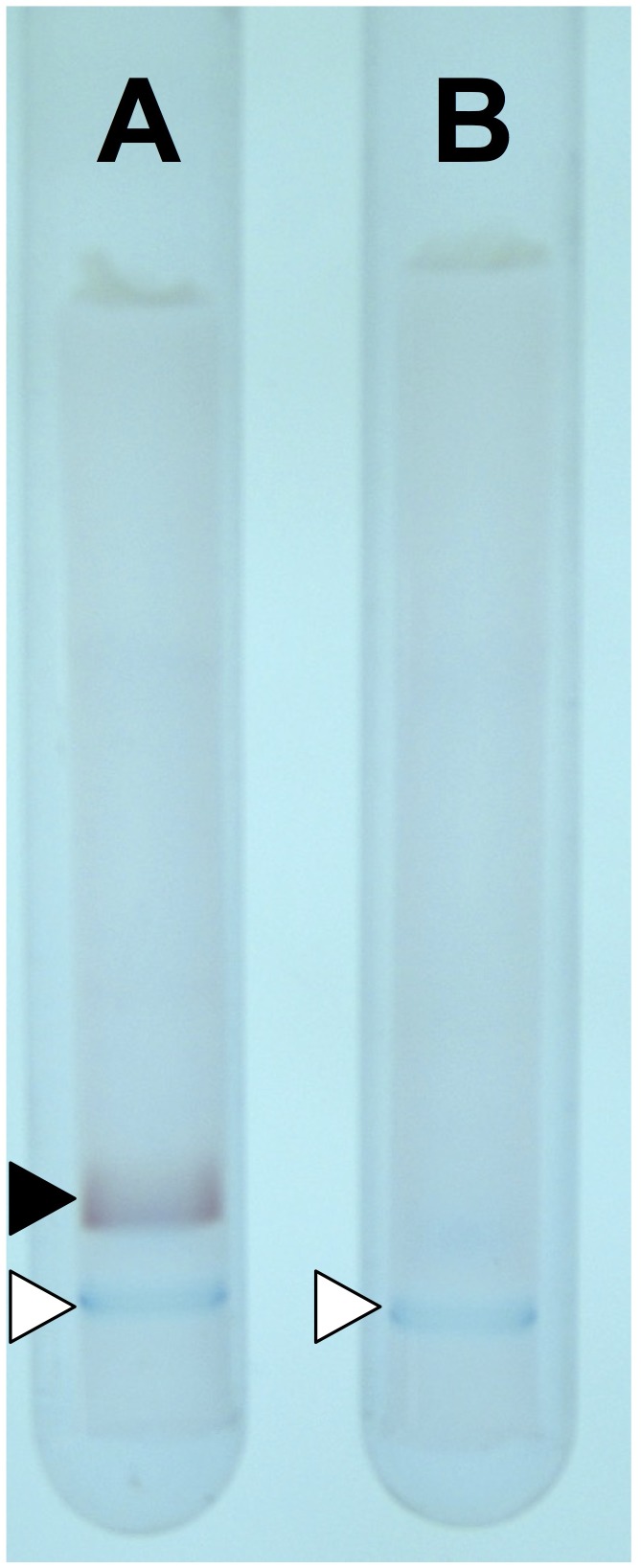
Activity staining with D-AADH. The staining was performed using D-isoleucine **(A)** and *meso*-diaminopimelate **(B)** as substrates. The activity band and color marker are inducted by filled and open triangles, respectively.

To create a thermostable D-AADH, five substitutions (Gln154Leu, Asp158Gly, Thr173Ile, Arg199Met, and His249Asn) were introduced into *U. thermosphaericus meso*-DAPDH using genetic engineering methods. The mutant enzyme was expressed in *E. coli*, after which the enzyme in the cell extract was purified using conventional methods. The parent enzyme acts specifically on *meso*-diaminopimelate and shows no activity toward D-isoleucine. On the other hand, native-PAGE of the purified mutant enzyme showed a single band when stained using an activity staining method with D-isoleucine as the electron donor, but not with *meso*-diaminopimelate as the donor (**Figure 1**). This result confirms that *meso*-DAPDH was successively changed to D-AADH. We next assessed the D-AADH activity of the mutant enzyme using several D- and L-amino acids. The mutant enzyme catalyzed the oxidative deamination of various D-amino acids in the presence of NADP^+^ (**Table 1**), as well as the reductive amination of various 2-oxo acids in the presence of NADPH and ammonia (Akita et al., 2012). Moreover, the substrate specificity of the mutant enzyme was low, in contrast to the high substrate specificity of *meso*-DAPDH. The mutant enzyme was also more thermostable than the parental *meso*-DAPDH; activity was retained, even after incubation at 65°C for 30 min in 50 mM potassium phosphate buffer (pH 7.2). The mutant enzyme was also stable over a wide pH range; more than 80% of the activity was retained at pHs between 5.5 and 10.5 (Akita et al., 2012). These features make this engineered enzyme potentially useful for application.

**Table 1:** Substrate specificities of D-AADH and its mutants.

Substrate	Oxidative deamination			

	His-tagged D-AADH Specific activity (μmol/min/mg)	Non-tagged D-AADH Specific activity (μmol/min/mg)	Asp94Ala Specific activity (μmol/min/mg)	Tyr224Phe Specific activity (μmol/min/mg)
D-Arginine	0.00738 ± 0.0002	0.722 ± 0.024	0.0815 ± 0.0027	0.337 ± 0.012
D-Histidine	-	0.0168 ± 0.0003	0.139 ± 0.003	0.0139 ± 0.0001
D-Isoleucine	0.193 ± 0.003	0.113 ± 0.002	1.70 ± 0.05	0.0763 ± 0.0016
D-Leucine	0.106 ± 0.004	0.191 ± 0.002	4.80 ± 0.11	0.0895 ± 0.0015
D-Lysine	0.299 ± 0.001	10.8 ± 0.1	1.32 ± 0.06	2.25 ± 0.02
D-Methionine	0.0178 ± 0.0009	0.213 ± 0.006	2.60 ± 0.11	0.173 ± 0.004
D-Norleucine	0.107 ± 0.002	0.419 ± 0.020	4.22 ± 0.10	0.315 ± 0.008
D-Phenylalanine	0.0106 ± 0.0002	0.099 ± 0.002	5.33 ± 0.14	0.0769 ± 0.0005
D-Tryptophan	-	0.0369 ± 0.0020	0.244 ± 0.006	0.0186 ± 0.0002
D-Valine	0.0142 ± 0.0008	0.121 ± 0.004	0.185 ± 0.005	0.0711 ± 0.0017

	**Reductive amination**			
	
2-Oxooctanoate	2.27 ± 0.06	13.2 ± 0.1	65.5 ± 0.8	11.7 ± 0.5
Phenylpyruvate	0.235 ± 0.005	1.93 ± 0.02	16.1 ± 0.3	1.67 ± 0.02
Pyruvate	0.0688 ± 0.0020	0.470 ± 0.029	0.131 ± 0.004	0.138 ± 0.003
2-Oxobutanoate	0.201 ± 0.002	2.47 ± 0.03	0.859 ± 0.052	0.85 ± 0.01
2-Oxohexanoate	1.03 ± 0.06	9.21 ± 0.13	35.0 ± 0.9	7.56 ± 0.17
2-Oxopentanoate	0.465 ± 0.015	4.72 ± 0.08	7.63 ± 0.20	3.44 ± 0.10
2-Oxo-3-methylbutanoate	0.279 ± 0.004	2.54 ± 0.05	1.04 ± 0.03	1.89 ± 0.01
2-Oxo-3-methylpentanoate	0.310 ± 0.007	2.23 ± 0.05	3.90 ± 0.06	2.25 ± 0.07
2-Oxo-4-methylpentanoate	0.624 ± 0.012	4.78 ± 0.11	15.7 ± 0.2	4.64 ± 0.06
2-Oxo-4-methylthio butanoate	0.624 ± 0.012	6.37 ± 0.09	25.0 ± 0.8	6.22 ± 0.12


## Production of D-BCAAs and Their Stable Isotope-Labeled Analogs

**FIGURE 2:**
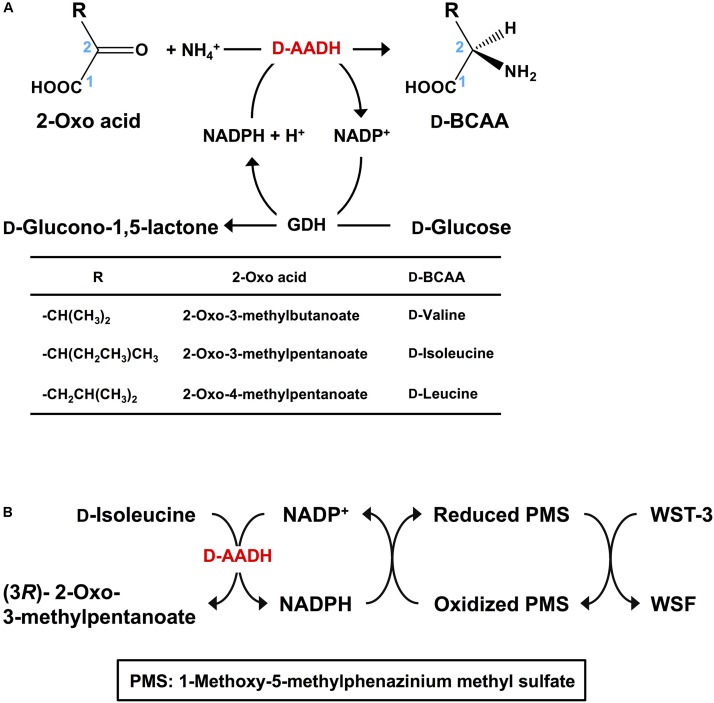
Application of D-AADH. **(A)** D-BCAA synthesis using D-AADH and GDH. DAADH catalyzes D-BCAA synthesis from appropriate 2-oxo acid, NH_4_^+^ and NADPH. NADPH is regenerated through glucose oxidation. **(B)** D-Isoleucine assay method employing two coupled reactions. NADPH is generated through D-isoleucine deamination. Water-soluble formazan is formed in parallel with the NADPH generation.

D-Branched-chain amino acids (D-BCAAs) such as D-isoleucine, D-leucine, and D-valine are known to exhibit special bioactivities (Kawagishi et al., 2004; Wang et al., 2004; Kolodkin-Gal et al., 2010), and are used as intermediates for several antibiotics (Velkov et al., 2010; Tempelaars et al., 2011; Doveston et al., 2012). For one step production of D-BCAAs from the corresponding 2-oxo acids, we developed a two enzyme-coupled system composed of D-AADH and glucose dehydrogenase (GDH) from the thermoacidophilic crenarchaeon *Sulfolobus tokodaii* (**Figure 2A**; Ohshima et al., 2003). Using this approach, a nearly 100% yield of optically pure D-leucine and D-valine from their oxo-analogs was achieved under optimized reaction conditions (Akita et al., 2014b). In the case of D-isoleucine, however, the maximal yield was about 50% because D-AADH catalyzes the conversion of (3*R*)-2-oxo-3-methylvalelate to D-isoleucine but not (3*S*)-2-oxo-3-methylvalerate to D-*allo*-isoleucine. GDH was used for NADPH regeneration from NADP^+^ through glucose oxidation. This system also can be utilized for effective production of five different D-BCAAs labeled with stable isotopes (Akita et al., 2014b). These include D-[1-^13^C, ^15^N]leucine, D-[1-^13^C]leucine, D-[^15^N]leucine, D-[^15^N]isoleucine, and D-[^15^N]valine (**Table 2**). All analyses with nuclear magnetic resonance, ultra-performance liquid chromatography, and time-of-flight mass spectrometry showed that neither L-BCAA nor unlabeled D-BCAA were present in the products. This system is the first enantioselective production of D-BCAAs labeled with stable isotopes (Akita et al., 2014b).

**Table 2:** Production of stable isotope-labeled D-BCAAs.

Product	Substrate	Optical purity (%)^a^	*m*/*z* [M+H]^+^	Chemical shift (ppm)	Yield (%)
D-[1-^13^C, ^15^N]Leucine	[1-^13^C]2-Oxo-4-methylpentanoate, [^15^N]NH_4_Cl	>99%	134.10	176.03 [^13^C] 47.32 [^15^N]	99 ± 1
D-[1-^13^C]Leucine	[1-^13^C]2-Oxo-4-methylpentanoate, NH_4_Cl	>99%	133.10	176.03	99 ± 1
D-[^15^N]Isoleucine	2-Oxo-3-methylpentanoate, [^15^N]NH_4_Cl	>99%	133.10	39.90	49 ± 1
D-[^15^N]Leucine	2-Oxo-4-methylpentanoate, [^15^N]NH_4_Cl	>99%	133.10	47.32	99 ± 0
D-[^15^N]Valine	2-Oxo-3-methylbutanoate, [^15^N]NH_4_Cl	>99%	119.08	43.09	99 ± 1


*^a^The values indicate the ratio of D-BCAAs to total D/L-BCAAs.*

Our system described here has several important features: one step production of D-amino acids and their analogs using coupled reactions of two thermostable dehydrogenases at high temperature (e.g., 60°C), which prevents microbial contamination of the reactor; extremely high optical purity of the products; practical synthesis of ^13^C- and/or ^15^N-labeled D-BCAAs; and an easily scalable production system (Ohshima and Soda, 2000).

## Spectrophotometric Assay of D-Isoleucine

D-Amino acids can be assayed using ultra-performance liquid chromatography (Ohmori et al., 2011), gas chromatography-mass spectrometry (Schurig, 2011), and liquid chromatography-tandem mass spectrometry (Konya et al., 2016). These methods are useful for simultaneous analysis of numerous kinds of D-amino acids. Indeed, the novel functions of D-amino acids in neurotransmitter transport (Nishikawa, 2011) and some human diseases (Katane and Homma, 2011) have been detected using these methods. However, these approaches require relatively expensive and sophisticated equipment. As an alternative, enzymatic methods have also been developed. For example, D-amino acid oxidase was utilized in colorimetric assays for several D-amino acids (Molla et al., 2012). Electrochemical analysis of D-proline (Tani et al., 2009) and spectrophotometric assay of D-serine (Suzuki et al., 2011) were developed with dye-linked D-proline dehydrogenase and D-serine dehydratase, respectively. However, no enzymatic assay for D-isoleucine has yet been reported. We therefore developed a simple and specific colorimetric system for assaying D-isoleucine using D-AADH. This enzymatic endpoint assay consisted of two successive reaction steps (**Figure 2B**): stoichiometric NADPH generation coupled to D-isoleucine deamination catalyzed by D-AADH followed by stoichiometric formation of water-soluble formazan (WSF) from water-soluble tetrazolium-3 (WST-3) coupled to oxidation of NADPH. After the reaction conditions were optimized, D-isoleucine was determined within a range of 1.0–50 μM (Akita et al., 2014a). We next examined the effect of three isoleucine analogs (100 μM) on enzymatic determination of D-isoleucine (1.0–50 μM). The D-AADH catalyzes oxidative deamination of D-isoleucine but not D-*allo*-isoleucine (a diastereomer of D-isoleucine), L-*allo*-isoleucine (a diastereomer of D-isoleucine), or L-isoleucine (an enantiomer of D-isoleucine) (Akita et al., 2012). Three isomers, D-*allo*-isoleucine, L-isoleucine, and L-*allo*-isoleucine gave no effect on the D-isoleucine determination. This enzymatic assay was thus useful for specific determination for D-isoleucine in the presence of other isoleucine isomers. Moreover, the assay was unaffected by the presence of alcohols (from distilled sprits) and several organic acids (from vinegar). We have thus developed the first enzymatic assay for D-isoleucine determination that appears suitable for industrial usage.

## Structural Comparison of *meso*-DAPDHs From *U. thermosphaericus* and Counterparts

Several three-dimensional structures of *meso*-DAPDHs from different sources have so far been solved. For the mesophilic *C. glutamicum* enzyme, these include the *meso*-DAPDH/NADP^+^ (Scapin et al., 1996) and *meso*-DAPDH/*meso*-diaminopimelate (Scapin et al., 1998) binary complexes as well as the *meso*-DAPDH/NADP^+^/inhibitor (Scapin et al., 1998) and *meso*-DAPDH/NADPH/inhibitor ternary complexes (Cirilli et al., 2000). In addition, the three-dimensional structure of thermophilic *S. thermophilum meso*-DAPDH has been solved as the apo form, the *meso*-DAPDH/NADP^+^ binary complex, and the *meso*-DAPDH/NADPH/*meso*-diaminopimelate ternary complex (Liu et al., 2014). These structural analyses enabled elucidation of the substrate and coenzyme recognition mechanisms of these enzymes. However, the structural features responsible for the high thermostability of thermophilic *meso*-DAPDH had not been evaluated. In addition, the specific factors responsible for the change in substrate specificity when D-AADH was created from *meso*-DAPDH by introducing five point mutations remained unknown. To address those issues, we determined the crystal structures of *meso*-DAPDH from *U. thermosphaericus*.

The apo form of *U. thermosphaericus meso*-DAPDH was assembled as a dimer, which was identical to the subunit assembly of the enzyme in solution (Akita et al., 2011). The monomer of the apo form consisted of three domains: a dinucleotide-binding domain, a dimerization domain, and a *C*-terminal domain (Akita et al., 2015). Structural studies of various thermophilic enzymes have so far revealed the molecular mechanisms underlying their higher thermostability in comparison to those of their mesophilic counterparts; the formation of ion pairs and their networks are responsible for the increase in thermostability (Hennig et al., 1995; Yip et al., 1995; Karshikoff and Ladenstein, 2001). Hydrophobic interactions also contribute to stabilization of the protein structures (Vieille and Zeikus, 2001; Bhuiya et al., 2005). In addition, conversion of polar and/or hydrophobic surface amino acids into charged residues increases thermostability (Fukuchi and Nishikawa, 2001). As mentioned, *U. thermosphaericus meso*-DAPDH shows much higher thermostability than the *C. glutamicum* enzyme, and is comparable to the thermostable *S. thermophilum meso*-DAPDH. Regarding subunit assembly, like the *C. glutamicum* enzyme, *U. thermosphaericus meso*-DAPDH is dimer and totally different from the hexameric *S. thermophilum* enzyme. There were fewer intrasubunit ion pairs within the *U. thermosphaericus* enzyme monomer (32–34) than *C. glutamicum meso*-DAPDH (50–53), but the number of intersubunit ion pairs was markedly higher in *U. thermosphaericus meso*-DAPDH (8 vs. 2). The total number of hydrophobic interactions (446 in subunit A, 447 in subunit B, and 159 intersubunit) in the *U. thermosphaericus* enzyme was much higher than in the *C. glutamicum* enzyme (357 in subunit A, 361 in subunit B, and 49 in intersubunit). We therefore consider that *U. thermosphaericus meso*-DAPDH achieves its higher thermostability through increases in the numbers of intersubunit ion pair networks and intra- and intersubunit hydrophobic interactions.

The total accessible surface area (ASA) of the *S. thermophilum meso*-DAPDH monomer (15,400 Å^2^) was comparable to that of the *C. glutamicum* enzyme monomer (15,600 Å^2^), and there was no significant difference in the number of hydrophobic interactions per monomer between the two enzymes. In *S. thermophilum* enzyme, the number of ion pairs per monomer is less than that in *C. glutamicum meso*-DAPDH. When the intersubunit interaction was compared, the number of ion pairs was not much different between the corresponding dimers of the two enzymes. By contrast, we observed fewer hydrophobic interactions (112–116) within the *S. thermophilum meso*-DAPDH dimer than the *U. thermosphaericus* enzyme (159). Overall, in the hexameric assembly of *S. thermophilum meso*-DAPDH, the greater numbers of intersubunit ion pairs (46) and hydrophobic interactions (467) were likely the main factors contributing to its high thermostability. Actually, a few thermophilic enzymes, including *Aeropyrum pernix* 2-deoxy-D-ribose-5-phosphate aldolase (Sakuraba et al., 2003) and *Thermococcus kodakaraensis* KOD1 ribulose 1,5-bisphosphate carboxylase/oxygenase (Maeda et al., 2002), achieve greater thermostability through oligomerization.

## Insight Into the Coenzyme and Substrate Recognition Mechanisms

**FIGURE 3:**
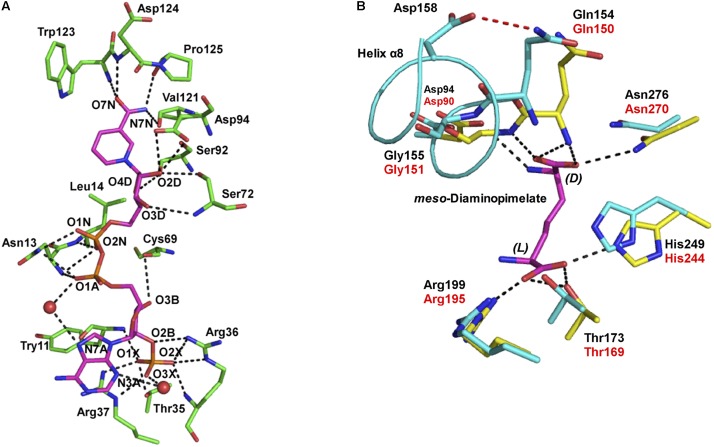
**(A)** The NADP^+^ binding site within *U. thermosphaericus meso*-DAPDH. The NADP^+^ molecule is shown in *magenta*. Water molecules are shown as *red* spheres. The networks of hydrogen bonds are shown as dotted lines. Oxygen, phosphorus, and nitrogen atoms are shown in *red, orange*, and *blue*, respectively. **(B)** The substrate binding site within *U. thermosphaericus meso*-DAPDH. The structure of *C. glutamicum meso*-DAPDH (*yellow* and red labels) is superimposed on that of *U. thermosphaericus meso*-DAPDH (*cyan* and black labels). A *meso*-diaminopimelate molecule is shown as a stick model in *magenta*. The hydrogen bond between Gln154 and Asp158 in *U. thermosphaericus meso*-DAPDH is shown as a dashed *red* line, and the networks of hydrogen bonds are shown as dotted lines. Oxygen and nitrogen atoms are shown in *red* and *blue*, respectively. These figures are from our previous study with some modification (Akita et al., 2015).

NADP^+^ differs from NAD^+^ in that it contains an additional phosphate group at the C2′ position of the adenine ribose. *U. thermosphaericus meso*-DAPDH shows high coenzyme specificity for NADP^+^, as NAD^+^ is inert as a coenzyme. To gain insight in the mechanism underlying this strict coenzyme specificity, we solved the structure of the NADP^+^-bound enzyme (Akita et al., 2015; **Figure 3A**). We found that fourteen amino acid residues interact with the NADP^+^ molecule within the structure of *U. thermosphaericus meso*-DAPDH. In particular, the side-chains of Thr35, Arg36, and Arg37 as well as the backbone nitrogens of Arg36 and Tyr11 form eight hydrogen bonds with the C2′-phosphate group of the adenine ribose. Upon the binding of NADP^+^ to the apo enzyme, the side chains of Arg36 and Arg37 were found to rotate to form hydrogen-bonds with the C2′-phosphate group of the adenine ribose (Akita et al., 2015). Thus, Arg36 and Arg37 may be strictly required for NADP^+^ recognition.

As discussed, *U. thermosphaericus* D-AADH does not act on *meso*-diaminopimelate but catalyzes reversible NADP^+^-dependent oxidative deamination of several D-amino acids. To assess the difference in substrate specificity between *meso*-DAPDH and D-AADH, the active site architecture of the *U. thermosphaericus meso*-DAPDH/NADP^+^ binary complex was compared with that of the *C. glutamicum meso*-DAPDH/*meso*-diaminopimelate binary complex. The residues that interact with *meso*-diaminopimelate in *C. glutamicum meso*-DAPDH (Asp90, Gln150, Gly151, Thr169, Arg195, His244, and Asn270) are completely conserved in the *U. thermosphaericus* enzyme as Asp94, Gln154, Gly155, Thr173, Arg199, His249, and Asn276, respectively (**Figure 3B**). Within the structure of the *C. glutamicum meso*-DAPDH/*meso*-diaminopimelate binary complex (Scapin et al., 1998), the side chain of Asp90, the main chain nitrogens of Gln150 and Gly151, and the side chain of Asn270 form five hydrogen bonds with the α-carboxylate of the D-amino acid center in the *meso*-diaminopimelate molecule. On the other hand, the side chains of Thr169, Arg195, and His244 also form four hydrogen bonds with the α-carboxylate of the L-amino acid center. When the thermostable D-AADH was created using *U. thermosphaericus meso*-DAPDH, Gln154Leu, Asp158Gly, Thr173Ile, Arg199Met, and His249Asn substitutions were introduced into the substrate recognition site. By introducing Thr173Ile, Arg199Met, and His249Asn substitutions, interactions between the enzyme and the α-carboxylate of the L-amino acid center were destroyed. As a result, D-AADH shows no activity toward *meso*-diaminopimelate. Moreover, the Thr173Ile and Arg199Met substitutions enhanced the hydrophobicity of the pocket around the L-amino acid center. Consequently, D-AADH shows high reactivity toward hydrophobic D-amino acids, such as D-cyclohexylalanine, D-isoleucine, and D-2-aminooctanoate. Within *U. thermosphaericus meso*-DAPDH, Gln154, and Asp158 are included in α-helix 8, with Gln154 being the *N*-terminal residue of the helix, and the side-chain of Gln154 forms a hydrogen bond with that of Asp158. The Gln154Leu and Asp158Gly substitutions cause this hydrogen bond to disappear, which enhances the flexibility of the *N*-terminal residues. This enhanced flexibility leads to greater reactivity toward several D-amino acids. Actually, the Asp154Gly substitution in *C. glutamicum* D-AADH, which corresponds to the Asp158Gly substitution in the *U. thermosphaericus* enzyme, also reportedly leads to broad substrate specificity for D-amino acids (Vedha-Peters et al., 2006).

## Improvement of D-AADH Catalytic Activity

Within the structure of the *C. glutamicum meso*-DAPDH/*meso*-diaminopimelate binary complex, one subunit is in an open conformation while the other is in a closed and substrate-bound conformation (Scapin et al., 1998). In addition, around the substrate-binding site, an ion-pair network is formed between the carboxyl group of the *C*-terminal residue Val320 and the side chains of surrounding residues, including His92, Asp120, and Arg128. This suggests the interaction around the carboxyl group of the *C*-terminal residue affects the catalytic activity of the enzyme. By contrast, *U. thermosphaericus* D-AADH includes additional *C*-terminal amino acid residues, Thr327 and Arg328 extend with a His-tag toward the corresponding region in the other subunit (Akita et al., 2015). This suggests the His-tag sequence within the *C*-terminal region of *U. thermosphaericus* D-AADH likely influences the enzyme’s activity. We therefore prepared non-tagged D-AADH and examined its catalytic properties. The non-tagged enzyme catalyzed NADP^+^-dependent deamination of several D-amino acids, and the activity was much higher than with the His-tagged enzyme (Hayashi et al., 2017; **Table 1**). In particular, the reaction rates for deamination of D-lysine, D-arginine, and D-norleucine by the non-tagged enzyme were much enhanced (Hayashi et al., 2017). For example, the specific activity toward D-lysine was about 36 times higher than that of the His-tagged D-AADH. This confirms that the interaction around the carboxyl group of the *C*-terminal residue plays a key role in the catalytic activity of the enzyme. The three-dimensional structure of the non-tagged D-AADH showed the presence of an ion-pair network at the carboxyl group of the *C*-terminal residue Leu326 and the side chains of the surrounding His96, Asp124, and Arg132 residues (Hayashi et al., 2017).

As the next step, with the aim of enabling development of additional applications, we sought to create a novel D-AADH whose substrate specificity differs from that of non-tagged D-AADH. Because we had succeeded in determining the structure of the apo form of non-tagged D-AADH but not the complexed form, we compared the active site architectures of apo non-tagged D-AADH with that of *S. thermophilum meso*-DAPDH in a ternary complex with NADPH and *meso*-diaminopimelate (Liu et al., 2014). Within the complexed *S. thermophilum* enzyme, the main-chain nitrogens of Met152 and Gly153 and the side chain nitrogen of Asn253 form three hydrogen bonds with the α-carboxylate in the D-amino acid center of *meso*-diaminopimelate. The side chain of Asp92 and the main-chain oxygen atom of Asp122 also form two hydrogen bonds with the α-amino group of the D-amino acid center. Moreover, the side chain of Asp92 forms a hydrogen bond with the side chain of Tyr205. Tyr205 is situated at the active-site entrance and is likely involved in substrate binding. The amino acid residues (Asp92, Asp122, Gly153, Tyr205, and Asn253) that interact with the D-center of *meso*-DAP in *S. thermophilum meso*-DAPDH were conserved in *U. thermosphaericus* DAADH (as Asp94, Asp124, Gly155, Tyr224, and Asn276, respectively). Although Met152 in the former was replaced by Leu154 in the latter, the backbone amide of Leu154 is situated in a position where it can interact with the D-center carboxyl of the substrate, as Met152 does. Asp94 and Tyr224 in *U. thermosphaericus* D-AADH were supposed to form a part of the substrate-binding pocket and play an important role in substrate binding. However, there had been no reported mutational analysis of these residues. We therefore prepared Asp94Ala and/or Tyr224Phe mutants of the non-tagged D-AADH and examined their D-amino acid deamination activity. The Asp94Ala mutant enzyme showed largely different substrate specificity than the parent enzyme (Hayashi et al., 2017; **Table 1**). In particular, the rate of D-phenylalanine deamination was markedly increased as the most preferable substrate, with specific activity 54 times higher than the parent enzyme. The reaction rates for deamination of D-leucine, D-norleucine, D-methionine, D-isoleucine, D-tryptophan, and D-histidine were also markedly elevated in comparison with the parent enzyme. By contrast, the Tyr224Phe mutant enzyme exhibited much less activity toward D-amino acids than the parent enzyme with almost all the substrates, especially for oxidative deamination, though the substrate spectrum was comparable. This suggests Tyr224 is essential for the proper catalytic activity, but is not very important for the substrate recognition.

**FIGURE 4:**
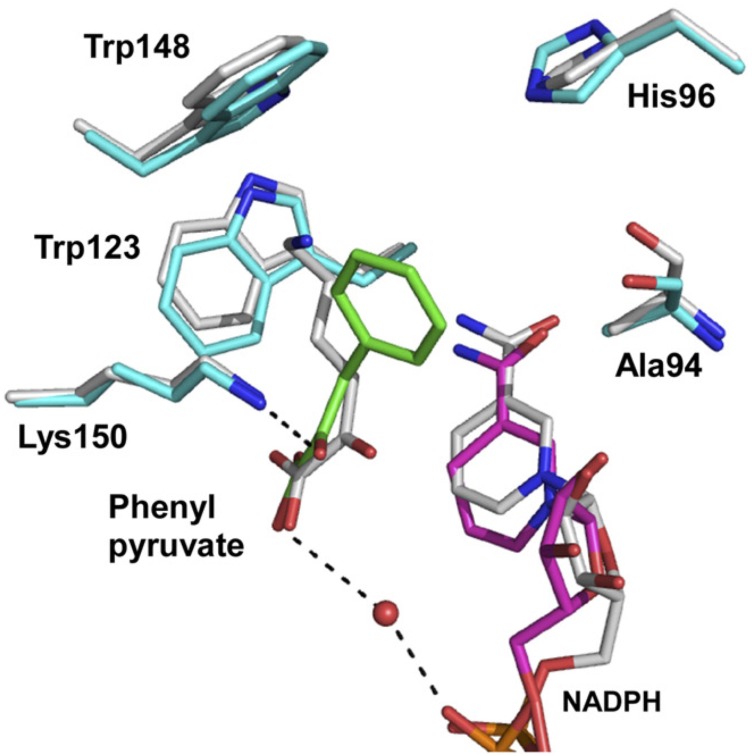
Proposed model for phenylpyruvate binding. The structures of the binding site within the NADPH/KACA/Asp94Ala-Tyr224Phe mutant enzyme complex, without and with phenylpyruvate bound, are shown in *white* and *cyan*, respectively. Phenylpyruvate and NADPH molecules in the phenylpyruvate-bound model are in *green* and *magenta*, respectively. The hydrogen bonds around the phenylpyruvate are shown as dotted lines. This figure is from our previous study with some modification (Hayashi et al., 2017).

To solve the structure of the enzyme with bound substrate/cofactor, in a preliminary study, we endeavored to co-crystalize the Asp94Ala mutant with NADP^+^/D-lysine, NADP^+^/D-phenylalanine, NADPH/D-lysine, or NADPH/D-phenylalanine. However, diffraction-quality crystals had not been prepared yet. We next constructed the Asp94Ala-Tyr224Phe double mutant and found that this enzyme showed much lower activity for oxidative deamination of D-lysine (0.48 μmol/min/mg) than the Asp94Ala or Tyr224Phe mutant. When we co-crystalized Asp94Ala-Tyr224Phe with NADP^+^/D-lysine, we succeeded in determining the three-dimensional structure of the NADPH/2-oxo-6-aminocapronate (KACA, the oxo acid analog of lysine)/enzyme ternary complex. Based on the orientation of KACA, we modeled the phenylpyruvate molecule into the active site of NADPH/KACA-bound Asp94Ala-Tyr224Phe and examined the factors responsible for the difference in substrate recognition (**Figure 4**). In this model, the carboxyl group and the C2′-carbonyl group of phenylpyruvate interact with the nicotinamide ribose phosphate and the side chain of Lys150, respectively. The phenyl group of the substrate interacts with the side chains of Ala94 (two interactions), Trp123 (five interactions), and Lys150 (eight interactions), in addition to stacking interactions with Trp148 and the nicotinamide ring. In the parent D-AADH, this phenyl group is supposed to be sterically hindered by the side chain of Asp94. Around the phenyl group, Trp123, Trp148, Lys150, and Ala94 form a large hydrophobic substrate-binding pocket. These observations suggest that the Asp94Ala substitution enlarges the substrate-binding pocket and enhances the hydrophobicity of the pocket around the side chain of the substrate. This may result in greater activity toward hydrophobic D-amino acids with bulky side chains, such as D-leucine, D-norleucine, D-tryptophan, and D-phenylalanine (**Table 1**).

## Conclusion

Through the work described above, we succeeded in finding the first known thermostable *meso*-DAPDH. The enzyme was isolated from *U. thermosphaericus* detected in compost, after which its gene sequence was determined, and a recombinant enzyme was produced. As the next step, we created a thermostable D-AADH from the *meso*-DAPDH by introducing five amino acid substitutions at the active site. This engineered enzyme catalyzed reversible deamination of various D-amino acids, making it possible to develop new one-step production methods for D-amino acids from 2-oxo acids and ammonia and enabling new production of stable isotope-labeled D-BCAAs. This is the first method for production of stable isotope-labeled D-BCAAs, and further development of enzymatic synthesis of other D-amino acids and analogs is expected. A spectrophotometric D-isoleucine assay using D-AADH will be highly useful as the first method for specific determination of D-isoleucine.

Our analysis of the functional structures of D-AADH made it possible to produce several functionally improved enzymes. One example is D-AADH with greatly increased activity achieved through removal of its *C*-terminal His tag, which in practical terms represents a reduction in cost, since fewer enzymes is needed for effective use. Another example is the alteration in substrate specificity caused by Asp94Ala substitution. This provided a novel D-AADH that exhibited higher reactivity toward D-phenylalanine, D-norleucine, and D-methionine. This mutant enzyme may open the way to production of the corresponding D-amino acids and isotope-labeled D-amino acids. These results clearly show that artificially designed stable enzymes offer great potential for future industrial applications. We are now planning to create additional useful D-AADH mutants exhibiting different substrate and coenzyme specificities. We anticipate that in the near future, thermostable engineered enzymes designed based on genomic and structural information and created through mutation will be used for industrial applications at least as often as naturally screened enzymes.

## Author Contributions

HA designed, conceived, and wrote the manuscript. JH helped in writing and editing. HS and TO critically reviewed, edited, and finalized the manuscript for submission.

## Conflict of Interest Statement

The authors declare that the research was conducted in the absence of any commercial or financial relationships that could be construed as a potential conflict of interest.

## References

[B1] Akita H. Doi K. Kawarabayasi Y. Ohshima T. (2012). Creation of a thermostable NADP^+^-dependent D-amino acid dehydrogenase from *Ureibacillus thermosphaericus* strain A1 *meso*-diaminopimelate dehydrogenase by site-directed mutagenesis. *Biotechnol. Lett.* 34 1693–1699. 10.1007/s10529-012-0952-1 22618239

[B2] Akita H. Fujino Y. Doi K. Ohshima T. (2011). Highly stable *mes*o-diaminopimelate dehydrogenase from an *Ureibacillus thermosphaericus* strain A1 isolated from a Japanese compost: purification, characterization and sequencing. *AMB Express* 1;43. 10.1186/2191-0855-1-43 22117688 PMC3293039

[B3] Akita H. Imaizumi Y. Suzuki H. Doi K. Ohshima T. (2014a). Spectrophotometric assay of D-isoleucine using an artificially created D-amino acid dehydrogenase. *Biotechnol. Lett.* 36 2245–2248. 10.1007/s10529-014-1597-z 24966047

[B4] Akita H. Seto T. Ohshima T. Sakuraba H. (2015). Structural insight into the thermostable NADP^+^-dependent *meso*-diaminopimelate dehydrogenase from *Ureibacillus thermosphaericus*. *Acta Crystol. D* 71 1136–1146. 10.1107/S1399004715003673 25945579

[B5] Akita H. Suzuki H. Doi K. Ohshima T. (2014b). Efficient synthesis of D-branched-chain amino acids and their labeled compounds with stable isotopes using D-amino acid dehydrogenase. *Appl. Microbiol. Biotechnol.* 98 1135–1143. 10.1007/s00253-013-4902-1 23661083

[B6] Araki T. Hidesaki T. Watanabe S. Nishida K. Nagahara K. Koito M. (2011). The Method of Manufacturing D-serine using an enzyme having D-serine synthesis activity. Japanese Patent No. JP2011172582A.

[B7] Becker J. Wittmann C. (2012). Systems and synthetic metabolic engineering for amino acid production–the heartbeat of industrial strain development. *Curr. Opin. Biotechnol.* 23 716–726. 10.1016/j.copbio.2011.12.025 22244788

[B8] Bhuiya M. W. Sakuraba H. Ohshima T. Imagawa T. Katunuma N. Tsuge H. (2005). The first crystal structure of hyperthermostable NAD-dependent glutamate dehydrogenase from *Pyrobaculum islandicum*. *J. Mol. Biol.* 345 325–337. 10.1016/j.jmb.2004.10.063 15571725

[B9] Born T. L. Blanchard J. S. (1999). Structure/function studies on enzymes in the diaminopimelate pathway of bacterial cell wall biosynthesis. *Curr. Opin. Chem. Biol.* 3 607–613. 10.1016/S1367-5931(99)00016-2 10508663

[B10] Chattopadhyay S. Raychaudhuri U. Chakraborty R. (2014). Artificial sweeteners-a review. *J. Food. Sci. Technol.* 51 611–621. 10.1007/s13197-011-0571-1 24741154 PMC3982014

[B11] Cirilli M. Scapin G. Sutherland A. Vederas J. C. Blanchard J. S. (2000). The three-dimensional structure of the ternary complex of Corynebacterium glutamicum diaminopimelate dehydrogenase-NADPH-L-2-amino-6-methylene-pimelate. *Protein Sci.* 9 2034–2037. 10.1110/ps.9.10.2034 11106178 PMC2144477

[B12] Doveston R. G. Steendam R. Jones S. Taylor R. J. (2012). Total synthesis of an oxepine natural product, (±)-janoxepin. *Org. Lett.* 14 1122–1125. 10.1021/ol300039x 22288766

[B13] Fukuchi S. Nishikawa K. (2001). Protein surface amino acid compositions distinctively differ between thermophilic and mesophilic bacteria. *J. Mol. Biol.* 309 835–843. 10.1006/jmbi.2001.4718 11399062

[B14] Gao X. Chen X. Liu W. Feng J. Wu Q. Hua L. (2012). A novel *meso*-Diaminopimelate dehydrogenase from *Symbiobacterium thermophilum*: overexpression, characterization, and potential for D-amino acid synthesis. *Appl. Environ. Microbiol.* 78 8595–8600. 10.1128/AEM.02234-12 23023754 PMC3502910

[B15] Hayashi J. Seto T. Akita H. Watanabe M. Hoshino T. Yoneda K. (2017). Structure-based engineering of an artificially generated NADP^+^-dependent D-amino acid dehydrogenase. *Appl. Environ. Microbiol.* 83:e00491-17. 10.1128/AEM.00491-17 28363957 PMC5440713

[B16] Hennig M. Darimont B. Sterner R. Kirschner K. Jansonius J. N. (1995). 2.0 Å structure of indole-3-glycerol phosphate synthase from the hyperthermophile *Sulfolobus solfataricus*: possible determinants of protein stability. *Structure* 3 1295–1306. 10.1016/S0969-2126(01)00267-2 8747456

[B17] Ivanov K. Stoimenova A. Obreshkova D. Saso L. (2013). Biotechnology in the production of pharmaceutical industry ingredients: amino acids. *Biotechnol. Biotechnol. Equip.* 27 3620–3626. 10.5504/BBEQ.2012.0134

[B18] Karshikoff A. Ladenstein R. (2001). Ion pairs and the thermotolerance of proteins from hyperthermophiles: a “traffic rule” for hot roads. *Trends Biochem. Sci.* 26 550–556. 10.1016/S0968-0004(01)01918-1 11551792

[B19] Katane M. Homma H. (2011). D-Aspartate–an important bioactive substance in mammals: a review from an analytical and biological point of view. *J. Chromatogr. Analyt. Technol. Biomed. Life Sci. B* 879 3108–3121. 10.1016/j.jchromb.2011.03.062 21524944

[B20] Kawagishi H. Hamajima K. Takanami R. Nakamura T. Sato Y. Akiyama Y. (2004). Growth promotion of mycelia of the matsutake mushroom *Tricholoma matsutake* by D-isoleucine. *Biosci. Biotechnol. Biochem.* 68 2405–2407. 10.1271/bbb.68.2405 15564685

[B21] Kolodkin-Gal I. Romero D. Cao S. Clardy J. Kolter R. Losick R. (2010). D-Amino acids trigger biofilm disassembly. *Science* 328 627–629. 10.1126/science.1188628 20431016 PMC2921573

[B22] Konya Y. Bamba T. Fukusaki E. (2016). Extra-facile chiral separation of amino acid enantiomers by LC-TOFMS analysis. *J. Biosci. Bioeng.* 121 349–353. 10.1016/j.jbiosc.2015.06.017 26321292

[B23] Leuchtenberger W. Huthmacher K. Drauz K. (2005). Biotechnological production of amino acids and derivatives: current status and prospects. *Appl. Microbiol. Biotechnol.* 69 1–8. 10.1007/s00253-005-0155-y 16195792

[B24] Liu W. Guo R. T. Chen X. Li Z. Gao X. Huang C. H. (2015). Structural analysis reveals the substrate-binding mechanism for the expanded substrate specificity of mutant *meso*-diaminopimelate dehydrogenase. *Chembiochem* 16 924–929. 10.1002/cbic.201402632 25754803

[B25] Liu W. Li Z. Huang C. H. Guo R. T. Zhao L. Zhang D. (2014). Structural and mutational studies on the unusual substrate specificity of *meso*-diaminopimelate dehydrogenase from *Symbiobacterium thermophilum.* *Chembiochem* 24 217–222. 10.1002/cbic.201300691 24339368

[B26] Maeda N. Kanai T. Atomi H. Imanaka T. (2002). The unique pentagonal structure of an archaeal Rubisco is essential for its high thermostability. *J. Biol. Chem.* 277 31656–31662. 10.1074/jbc.M203117200 12070156

[B27] Martínez-Rodríguez S. Martínez-Gómez A. I. Rodríguez-Vico F. Clemente-Jiménez J. M. Las Heras-Vázquez F. J. (2010). Natural occurrence and industrial applications of D-amino acids: an overview. *Chem. Biodivers.* 7 1531–1548. 10.1002/cbdv.200900245 20564568

[B28] Misono H. Ogasawara M. Nagasaki S. (1986a). Characterization of *meso*-diaminopimelate dehydrogenase from *Corynebacterium glutamicum* and its distribution in bacteria. *Agric. Biol. Chem.* 50 2729–2734. 10.1271/bbb1961.50.2729

[B29] Misono H. Ogasawara M. Nagasaki S. (1986b). Purification and properties of *meso*-diaminopimelate dehydrogenase from *Brevibacterium* sp. *Agric. Biol. Chem.* 50 1329–1330. 10.1080/00021369.1986.10867567

[B30] Misono H. Soda K. (1980). Properties of *meso*-α,ε-diaminopimelate D-dehydrogenase from *Bacillus sphaericus*. *J. Biol. Chem.* 255 10599–10605. 7430138

[B31] Misono H. Togawa H. Yamamoto T. Soda K. (1979). *meso*-α,ε-diaminopimelate D-dehydrogenase: distribution and the reaction product. *J. Bacteriol.* 137 22–27. 762012 10.1128/jb.137.1.22-27.1979 PMC218413

[B32] Molla G. Piubelli L. Volontè F. Pilone M. S. (2012). Enzymatic detection of D-amino acids. *Methods Mol. Biol.* 794 273–289. 10.1007/978-1-61779-331-8_18 21956570

[B33] Nishikawa T. (2011). Analysis of free D-serine in mammals and its biological relevance. *J. Chromatogr. B Analyt. Technol. Biomed. Life Sci.* 879 3169–3183. 10.1016/j.jchromb.2011.08.030 21992750

[B34] Ohmori T. Mutaguchi Y. Yoshikawa S. Doi K. Ohshima T. (2011). Amino acid components of lees in salmon fish sauce are tyrosine and phenylalanine. *J. Biosci. Bioeng.* 112 256–258. 10.1016/j.jbiosc.2011.05.009 21658995

[B35] Ohshima T. Ito Y. Sakuraba H. Goda S. Kawarabayasi Y. (2003). The *Sulfolobus tokodaii* gene ST1704 codes highly thermostable glucose dehydrogenase. *J. Mol. Catal. B Enzym.* 23 281–289. 10.1016/S1381-1177(03)00091-2

[B36] Ohshima T. Soda K. (2000). “Amino acid dehydrogenases and their applications,” in *Stereoselective Biocatalysis*, ed. Patel R. N. (New York, NY: Marcel Dekker), 877–902.

[B37] Sakuraba H. Tsuge H. Shimoya I. Kawakami R. Goda S. Kawarabayasi Y. (2003). The first crystal structure of archaeal aldolase. Unique tetrameric structure of 2-deoxy-D-ribose-5-phosphate aldolase from the hyperthermophilic archaea *Aeropyrum pernix*. *J. Biol. Chem.* 278 10799–10806. 10.1074/jbc.M212449200 12529358

[B38] Scapin G. Cirilli M. Reddy S. G. Blanchard J. S. (1998). Substrate and inhibitor binding sites in *Corynebacterium glutamicum* diaminopimelate dehydrogenase. *Biochemistry* 37 3278–3285. 10.1021/bi9727949 9521647

[B39] Scapin G. Reddy S. G. Blanchard J. S. (1996). Three-dimensional structure of *meso*-diaminopimelic acid dehydrogenase from *Corynebacterium glutamicum*. *Biochemistry* 35 13540–13551. 10.1021/bi961628i 8885833

[B40] Schmid A. Hollmann F. Park J. B. Bühler B. (2002). The use of enzymes in the chemical industry in Europe. *Curr. Opin. Biotechnol.* 13 359–366. 10.1016/S0958-1669(02)00336-1 12323359

[B41] Schurig V. (2011). Gas chromatographic enantioseparation of derivatized α-amino acids on chiral stationary phases–past and present. *J. Chromatogr. B* 879 3122–3140. 10.1016/j.jchromb.2011.04.005 21530421

[B42] Silber J. Kramer A. Labes A. Tasdemir D. (2016). From discovery to production: biotechnology of marine fungi for the production of new antibiotics. *Mar. Drugs* 14:E137. 10.3390/md14070137 27455283 PMC4962027

[B43] Suzuki C. Murakami M. Yokobori H. Tanaka H. Ishida T. Horiike K. (2011). Rapid determination of free D-serine with chicken D-serine dehydratase. *J. Chromatogr. B* 879 3326–3330. 10.1016/j.jchromb.2011.07.028 21840271

[B44] Tani Y. Itoyama Y. Nishi K. Wada C. Shoda Y. Satomura T. (2009). An amperometric D-amino acid biosensor prepared with a thermostable D-proline dehydrogenase and a carbon nanotube-ionic liquid gel. *Anal. Sci.* 25 913–923. 10.2116/analsci.25.919 19609033

[B45] Tempelaars M. H. Rodrigues S. Abee T. (2011). Comparative analysis of antimicrobial activities of valinomycin and cereulide, the *Bacillus cereus* emetic toxin. *Appl. Environ. Microbiol.* 77 2755–2762. 10.1128/AEM.02671-10 21357430 PMC3126370

[B46] Vedha-Peters K. Gunawardana M. Rozzell J. D. Novick S. J. (2006). Creation of a broad-range and highly stereoselective D-amino acid dehydrogenase for the one-step synthesis of D-amino acids. *J. Am. Chem. Soc.* 128 10923–10929. 10.1021/ja0603960 16910688 PMC2533268

[B47] Velkov T. Thompson P. E. Nation R. L. Li J. (2010). Structure–activity relationships of polymyxin antibiotics. *J. Med. Chem.* 53 1898–1916. 10.1021/jm900999h 19874036 PMC2907661

[B48] Vieille C. Zeikus G. J. (2001). Hyperthermophilic enzymes: sources, uses, and molecular mechanisms for thermostability. *Microbiol. Mol. Biol. Rev.* 65 1–43. 10.1128/MMBR.65.1.1-43.2001 11238984 PMC99017

[B49] Wakayama M. Yoshimune K. Hidese Y. Moriguchi M. (2003). Production of D-amino acids by *N*-acyl-D-amino acid amidohydrolase and its structure and function. *J. Mol. Catal. B Enzym.* 23 71–85. 10.1016/S1381-1177(03)00074-2

[B50] Wang W. Liu G. Yamashita K. Manabe M. Kodama H. (2004). Characteristics of prolinase against various iminodipeptides in erythrocyte lysates from a normal human and a patient with prolidase deficiency. *Clin. Chem. Lab. Med.* 42 1102–1108. 10.1515/CCLM.2004.227 15552267

[B51] Yip K. S. Stillman T. J. Britton K. L. Artymiuk P. J. Baker P. J. Sedelnikova S. E. (1995). The structure of *Pyrococcus furiosus* glutamate dehydrogenase reveals a key role for ion-pair networks in maintaining enzyme stability at extreme temperatures. *Structure* 15 1147–1158. 10.1016/S0969-2126(01)00251-9 8591026

